# Ecological Analysis of the Helminth Community and Its Relationship with the Physiological State in the Montane Water Vole, *Arvicola scherman* (Shaw, 1801), in NW Spain

**DOI:** 10.3390/ani16081162

**Published:** 2026-04-10

**Authors:** Roser Adalid, Carles Feliu, Aitor Somoano, Marcos Miñarro, Jacint Ventura, Jordi Miquel, Màrius Vicent Fuentes

**Affiliations:** 1 Secció de Parasitologia, Departament de Biologia, Sanitat i Medi Ambient, Facultat de Farmàcia i Ciències de l’Alimentació, Universitat de Barcelona, Av. Joan XXIII, s/n, 08028 Barcelona, Spain; roseradalid@ub.edu (R.A.); cfeliu@ub.edu (C.F.); jordimiquel@ub.edu (J.M.); 2 Institut de Recerca de la Biodiversitat (IRBio), Universitat de Barcelona, Av. Diagonal, 645, 08028 Barcelona, Spain; 3 Servicio Regional de Investigación y Desarrollo Agroalimentario (SERIDA), Ctra. AS-267, PK 19, 33300 Villaviciosa, Spain; aitor.somoanogarcia@asturias.org (A.S.); marcos.minarroprado@asturias.org (M.M.); 4 Departament de Biologia Animal, Biologia Vegetal i Ecologia, Facultat de Biociències, Campus de Bellaterra, Universitat Autònoma de Barcelona, 08193 Cerdanyola del Vallès, Spain; jacint.ventura.queija@uab.cat; 5 Naturals Sciences Museum of Granollers, C/Palaudàries, 102, 08402 Granollers, Spain; 6 Parasites and Health Research Group, Departament de Farmàcia i Tecnologia Farmacèutica i Parasitologia, Facultat de Farmàcia i Ciències de l’Alimentació, Universitat de Valencia, Av. Vicent Andrés Estellés, 46100 Burjassot, Spain

**Keywords:** body condition, helminth parasites, biotic and abiotic factors, voles, *Arvicola scherman*, apple orchards, agricultural pest

## Abstract

The montane water vole, *Arvicola scherman*, is a fossorial rodent that lives underground in grasslands, pastures and meadows in the mountain ranges of southwestern and central Europe. It feeds mainly on grasses, roots, and bulbs, causing considerable economic damage to agriculture. Consequently, it is recognised as one of the most important pest vole species in European agroecosystems. The dynamics of these pest populations may be affected by interactions with their parasites. For this reason, an helminthoecological study was carried out in Asturias (NW Spain), analysing a total of 815 montane water voles, 464 (56.9%) of which were parasitised by at least one of the six helminth species detected: *Hydatigera taeniaeformis* s.l. larvae (9%), *Aonchotheca wioletti* (0.1%), *Eucoleus bacillatus* (0.4%), *Trichuris arvicolae* (7%), *Carolinensis minutus* (30%) and *Syphacia nigeriana* (12%). The helminth community found was compared with that reported from *A. scherman* analysed in other locations of the Iberian Peninsula. This study also analyses the influence of intrinsic and extrinsic factors on the prevalence and abundance of the helminth component species, with host age and body condition being the most influential determinants. *H. taeniaeformis* s.l. and *T. arvicolae* are postulated as potential regulators of the analysed population, a pest in crops from NW Spain.

## Simple Summary

The montane water vole, *Arvicola scherman*, is a rodent that lives underground in mountain grasslands and farmlands across parts of Europe. It mainly feeds on grass and roots and can cause serious damage to agriculture, especially apple orchards, making it an important pest species. A study in Asturias (NW Spain) examined helminth parasites in this vole species. A total of 815 individuals was analysed, with about 57% found to be parasitised by at least one of six helminth species. The most common parasites were *Carolinensis minutus* and *Syphacia nigeriana*. The helminth community found in these voles was compared to that reported from other *A. scherman* populations analysed in other locations of the Iberian Peninsula. This study also analyses how biological and environmental factors can affect helminth parasitation. Host age and body condition were the most important factors influencing infection levels. Moreover, two helminth species, *Hydatigera taeniaeformis* s.l. and *Trichuris arvicolae*, could regulate vole population size in the studied area.

## 1. Introduction

The montane water vole, *Arvicola scherman*, is a fossorial rodent belonging to the family Cricetidae, subfamily Arvicolinae, a lineage characterised by a rapid evolutionary radiation in temperate and cold ecosystems of the Northern Hemisphere [1]. The taxonomy of *Arvicola* has long been controversial. Populations formerly assigned to the morphotype *scherman* were traditionally considered a subspecies of *Arvicola terrestris*, a species name that is no longer regarded as valid. In the recent literature, Iberian populations are referred to under different names, including *A. scherman*, *Arvicola amphibius* and *Arvicola monticola* [2,3,4,5,6]. Following Musser et al. (2005) [2] and the latest taxonomic evidence [7], the name *A. scherman* is adopted here, emphasising its fossorial locomotion and predominantly subterranean lifestyle.

The montane water vole is distributed across the main mountain ranges of southwestern and central Europe, including the Cantabrian Mountains, the Pyrenees, the Alps and the Carpathians [3]. In the Iberian Peninsula, two subspecies are currently recognised: *A. scherman monticola*, restricted to the Pyrenees, and *A. scherman cantabriae*, confined to the Cantabrian region [6,8]. The species has a strictly herbivorous diet, consuming both aerial and underground plant parts [9]. Since *A. scherman* has a high reproductive potential [10], it can reach population densities of up to 1000 voles/ha [11], making it a critical prey species for numerous raptors and predatory mammals within agroecosystems [12]. Given its high population densities in grasslands and agricultural systems, it can cause severe damage to crops, leading to significant economic losses [11,13]. Hence, the montane water vole is considered an agricultural pest in several European countries, such as Spain, where population control measures are officially recommended [14]. Given the recurrent population outbreaks of *A. scherman* in the northwestern Iberian Peninsula [13,15], and considering that parasitism can affect the viability of rodent populations [16] and, consequently, their impact as agricultural pests, as well as generate cascading effects across the trophic web [12], investigating the diversity, prevalence, and ecological roles of their helminthfauna is both ecologically and practically relevant.

Endoparasites, particularly helminths, are widely recognised as key components of natural systems regulating host populations, as they can influence host survival, reproductive output and the physiological condition of their hosts [16,17,18]. In arvicoline rodents, the composition and structure of helminth communities are shaped by a combination of extrinsic and intrinsic factors, including host density, habitat characteristics, seasonality, and individual traits such as age, sex, and body condition [19,20]. Previous studies on *Arvicola* taxa have reported relatively diverse helminth assemblages, typically dominated by gastrointestinal nematodes and accompanied by cestodes, but most Iberian studies have focused on northeastern regions [21,22] where *A. scherman monticola* is found. However, information on the westernmost populations, corresponding to *A. scherman cantabriae*, remains scarce. Consequently, the diversity, structure and ecological drivers of helminth communities in northwestern Iberian populations—particularly during demographic outbreaks—remain poorly understood.

Moreover, despite its relevance for assessing parasite-mediated effects on population regulation, the relationship between helminth burden and host body condition—a key proxy of physiological status—has rarely been evaluated in wild populations. Helminth infections are known to impose chronic and sub-lethal costs on hosts, affecting energy allocation, immune regulation and overall physiological condition [16,23,24]. In wild mammal populations, higher parasite burdens have been associated with reduced body condition and potentially compromised fitness [25]. In arvicoline rodents, parasite species such as *Trichuris arvicolae* and *Hydatigera taeniaeformis* s.l. have been suggested to influence host reproductive parameters and could act as natural regulators of population growth [26]. Higher parasite loads may reduce host body condition, increasing vulnerability to predation and potentially enhancing natural biological control processes [27]. Moreover, decreases in body condition can lead to negative consequences for biological fitness and may interact with multiannual density phases observed in rodent population dynamics [10].

In this context, *A. scherman* represents a suitable model to evaluate how helminth parasitism may affect the host body condition and how such effects could be indirectly associated with processes influencing population density in overabundant rodent populations. This study aims to (i) characterise the helminth community infecting *A. scherman* populations in the northwestern Iberian Peninsula; (ii) compare these findings with previous data from other Iberian populations; (iii) analyse spatial and seasonal variation in parasite communities; and (iv) assess the relationship between helminth burden and host body condition as an indicator of potential ecological and sanitary impacts.

## 2. Materials and Methods

### 2.1. Study Area

**Figure 1:**
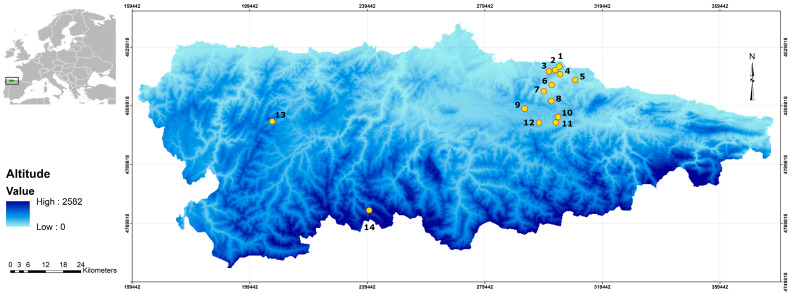
Map of Asturias, in northwest Spain, showing the capture locations of *Arvicola scherman* analysed. 1. Santa Marina; 2. Llata; 3. Oles; 4. La Rozada; 5.; Priesca; 6. SERIDA; 7. Camoca; 8. Poreño; 9. Fresnu; 10. Fresnadiello; 11. Ceceda; 12. Vegadali; 13. Valle del Lago; 14. La Fajera.

Asturias is located on the north-central coast of Spain (43°30′ N, 5°30′ W) (Figure 1). This region is characterised by temperate oceanic and temperate hyperoceanic bioclimates, with abundant rainfall spread fairly throughout the year and mild temperatures in both summer and winter with a low risk of frost and snowfall [28]. Asturias is characterised by small agricultural plots separated by hedgerows and woodlands, with an irregular topography of smooth hills and valleys [29]. The fertile soil added to these weather conditions favours the establishment of an evergreen and dense vegetal cover in orchards all year round [30,31]. This allows *A. scherman* populations to sustain a markedly elevated reproductive potential, with no seasonal suppression or interruption of reproductive function across the annual cycle [10,32]. All specimens of *A. scherman* analysed in this study originated from apple orchards in this area, where this rodent is a key pest.

### 2.2. Zoological and Helminthological Procedures

**Figure 2:**
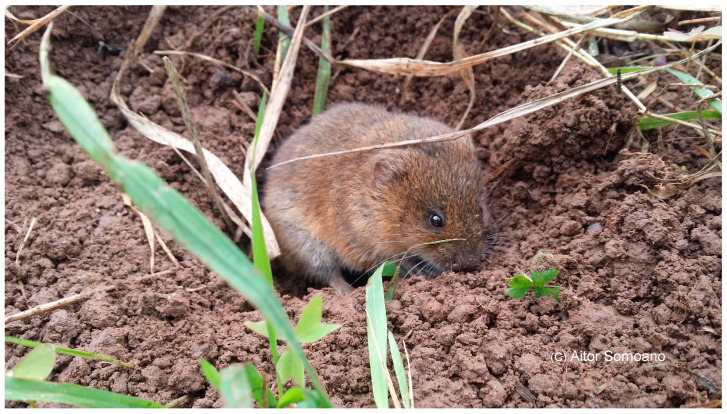
An individual of *Arvicola scherman* in its natural habitat in one of the locations prospected.

A total of 815 specimens of *A. scherman cantabriae* (Figure 2), obtained from the collection of SERIDA (Servicio Regional de Investigación y Desarrollo Agroalimentario, Asturias), was helminthologically analysed. The sample was collected during two consecutive years (January 2011 to January 2013) in apple orchards from 14 Asturian localities (Figure 1). The recommendations of the Directive of the European Parliament and of the Council on the Protection of Animals Used for Scientific Purposes [33] were followed in the field work.

Of the total individuals analysed, 401 males and 414 females, 427 were captured in 2011, 372 in 2012 and 16 in 2013. Concerning the year of capture, in view of the small number of voles captured in 2013, with all of them captured in January, the analysis of this factor considered only two types of values, those of voles captured in 2011 and those of voles captured in 2012 and 2013 together (n = 388).

Specimens were previously assigned to six classes of relative age (0–5) [32] according to the criteria by Ventura (1993) [34]. In the present study, this classification was simplified as follows: juvenile individuals, classes 0 and 1; preadult individuals, classes 2 and 3; and adult individuals, classes 4 and 5. According to this, the sample was composed of: 93 juvenile, 185 preadult and 537 adult specimens (Table 1). Seasons were defined as: winter (January–March), spring (April–June), summer (July–September) and autumn (October–December). Following this distribution by month, 169 captures took place in winter, 227 in spring, 197 in summer and 222 in autumn (Table 2).

All specimens included in the analyses were dissected in the SERIDA facilities under standardised laboratory conditions. Viscera were extracted for the parasitological study in the parasitology laboratory of the University of Barcelona. All helminths found were preserved in 70% ethanol. Some nematodes of different species, as well as some scolices of cestodes, were mounted in slides with Amman lactophenol to clear these helminths. To identify selected helminths at the specific level, their morphology and morphometry were analysed and compared with data reported in the most relevant descriptions and findings in the scientific literature.

### 2.3. Helminth Community Analysis

The analysis of the **helminth community composition and structure** of the montane water vole was carried out by considering each particular life cycle and calculating the prevalence, mean abundance, median intensity and range as well as total number of helminths, calculated following standard parasitological definitions according to Bush et al. (1997) [35]. Prevalence represents the percentage of infected hosts, while mean abundance corresponds to the average number of individuals of a particular helminth species per examined hosts, and mean intensity is the same but per parasitised hosts only.

The analysis of the **helminth community components** was conducted by calculating the frequency of occurrence of the number of helminth species, the abundance index (AI) (calculated excluding those parasite species for which *A. scherman* acts as intermediate host) and the frequency distribution of helminths. The frequency of species distribution, a parameter distinct from species richness, refers to the infracommunities of the montane water vole. This parameter is expressed as a percentage, which shows the frequency of the number of species that make up the infracommunities and, therefore, the distribution of the entire group of species that make up the helminth community within the host population. The abundance index was calculated to determine the ecological importance of each helminth species within the community. Based on AI values, species were classified as dominant (AI > 1), co-dominant (0.1 ≤ AI ≤ 1), successful immigrant (0 < AI < 0.1), or unsuccessful immigrant (AI = 0) [36,37].

The frequency distribution pattern of helminth species was assessed using the Lefkovitch index (L) [38], where

L = (1/45) tang^−1^ (variance/mean) − 1

This index describes the distribution of parasites among hosts, ranging from uniform (−1) to random (0) to aggregated (+1).

The **diversity/uniformity of the helminth community** was assessed using: the Shannon index (H’)—its value ranges from 0 to 5 and is based on information obtained from assessing the helminth species richness [39,40]; Simpson index (D’)—its value ranges from 0 to 1, and it is a dominance index weighted according to the abundance of the most common helminth species [41], expressed as 1-D’ [40]; Berger–Parker index (d)—its value ranges from 0 to 1, and it is also an assessment of dominance that expresses the importance of the most abundant helminth species [42,43], expressed as 1-d [40]; and Shannon evenness index (E)—its value ranges from 0 to 1, and it is an assessment of uniformity that considers, like the Shannon index, that all species present in the helminth community have been considered in the sample [40,44].

The **helminth infracommunity diversity** was assessed using the analysis of the number of helminths per host (mean abundance), number of helminth species (species richness), Brillouin index (HB), Brillouin index for infected hosts only and percentage of infected hosts. This is the only index used to measure the specific diversity of helminth infracommunities, and like the Shannon index, it weights species richness; its value rarely exceeds 4.5 [39,40].

The role played by intrinsic (host sex and age) and extrinsic (climate data, year and season of capture) factors (independent variables) in determining the helminth community dependent variables (species richness—the number of helminths species; % of parasitation; and the worm burden—helminth abundance) was statistically analysed. Climate data, related to temperature (mean daily temperature calculated for each season) and precipitation (mean daily rainfall calculated for each season and cumulative rainfall during each season) (Table 3), were obtained from the nearest climate stations (AEMET—Spanish Meteorological Agency). Values of climate variables, belonging to the year before capture, are related to both variables by means of the Spearman correlation coefficient (r_s_); prevalence was previously transformed logarithmically using log (x/(1 − x)). The influence of year and season of capture, and host sex and age on prevalence was analysed using binary logistic regression (BLR), while their influence on the other dependent variables was analysed by means of standard non-parametric tests, i.e., the Mann–Whitney (U) and Kruskal–Wallis (H) tests.

### 2.4. Association Between Body Condition and Parasite Burden

The Body Condition Index (BCI) was estimated following the method proposed in [45], which is based on standardised major axis (SMA) regression. This approach uses the Scaled Mass Index (SMI) to adjust an individual’s body mass to a fixed value of a linear body measurement, capturing the allometric relationship between mass and length. The SMI is calculated as

$$\mathrm{SMI}=m_{i}{\left(\frac{L_{0}}{L_{i}}\right)}^{b_{SMA}}$$

where $m_{i}$ and $L_{i}$ represent the body mass and the head–body length of individual i, $L_{0}$ is a reference length (the arithmetic mean of head–body length for the study population), and $b_{SMA}$ is the scaling exponent obtained from the SMA regression of ln(mass) against ln(length).

Reference data for this calculation came from specimens of *A. scherman* previously analysed by Somoano et al. (2017) [32]. This method standardises body mass relative to structural size, improving comparability among individuals with different body proportions [45,46]. The reference population was collected under conditions suggesting good health, abundant food, and no evident environmental stress, so body condition scores likely reflect physiological status rather than nutritional limitations.

To assess the relationship between the physiological state and parasite burden, associations between the BCI and several parasitological descriptors were analysed, including parasite species richness, the abundance of individual parasite species, and aggregated measures of parasitism. Initially, the direction and strength of relationships between BCI and each parasitological variable were explored using Spearman rank correlations. As parasite abundance data did not meet normality assumptions, non-parametric statistical approaches were subsequently applied to test for differences among body condition groups. Individuals were classified into BCI tertiles representing high, medium and low body condition (high: mean = 24.85, range = 14.26–53.67; medium: mean = 6.69, range = −3.49–14.24; low: mean = −23.76, range = −70.03 to −3.64). Global differences among tertiles were evaluated using Kruskal–Wallis tests. When significant effects were detected, pairwise post hoc comparisons were conducted using Mann–Whitney tests, with *p*-values adjusted for multiple comparisons using the Holm–Bonferroni correction.

SPSS 26.0—IBM for Windows and Python Language Reference (Version 3.11) were the software packages used for statistical analyses. Statistical significance was established at *p* < 0.05.

## 3. Results

### 3.1. Helminth Community Composition and Structure

A total of 464 (57%) individuals of montane water voles was found to be parasitised by at least one of the six helminth species identified: one cestode, *Hydatigera taeniaeformis* s.l. larvae (Taeniidae), and five nematodes, *Aonchotheca wioletti*, *Eucoleus bacillatus* (Capillariidae), *Trichuris arvicolae* (Trichuridae), *Carolinensis minutus* (Trichostrongylidae) and *Syphacia nigeriana* (Oxyuridae). *Carolinensis minutus* was the most prevalent (30.0%) and the most abundant (3.12) species, while *S. nigeriana* presented the highest parasitation values, with a mean intensity of 5 and a maximum range of 348 individuals (Table 4). This is the first time that *C. minutus* has been found in *A. scherman* from the Iberian Peninsula.

#### 3.1.1. *Hydatigera taeniaeformis* s.l. (Batsch, 1786) Larvae (Figure 3)

The larval stage of this Taenidae tapeworm is a globular cyst of 5–12 mm in diameter, found on the liver surface. Inside this cyst there is the larval stage named strobilicercoid, the scolex of which has a rostellum with hooks of two different sizes [47]; in the larvae found in *A. scherman*, the number of hooks varied between 28 and 36, with large hooks measuring 443–484 µm and small ones measuring 238–299 µm. The intermediate hosts are infected after the ingestion of eggs released with the faeces of the definitive hosts, mainly cats, contaminating the soil and vegetables.

**Figure 3:**
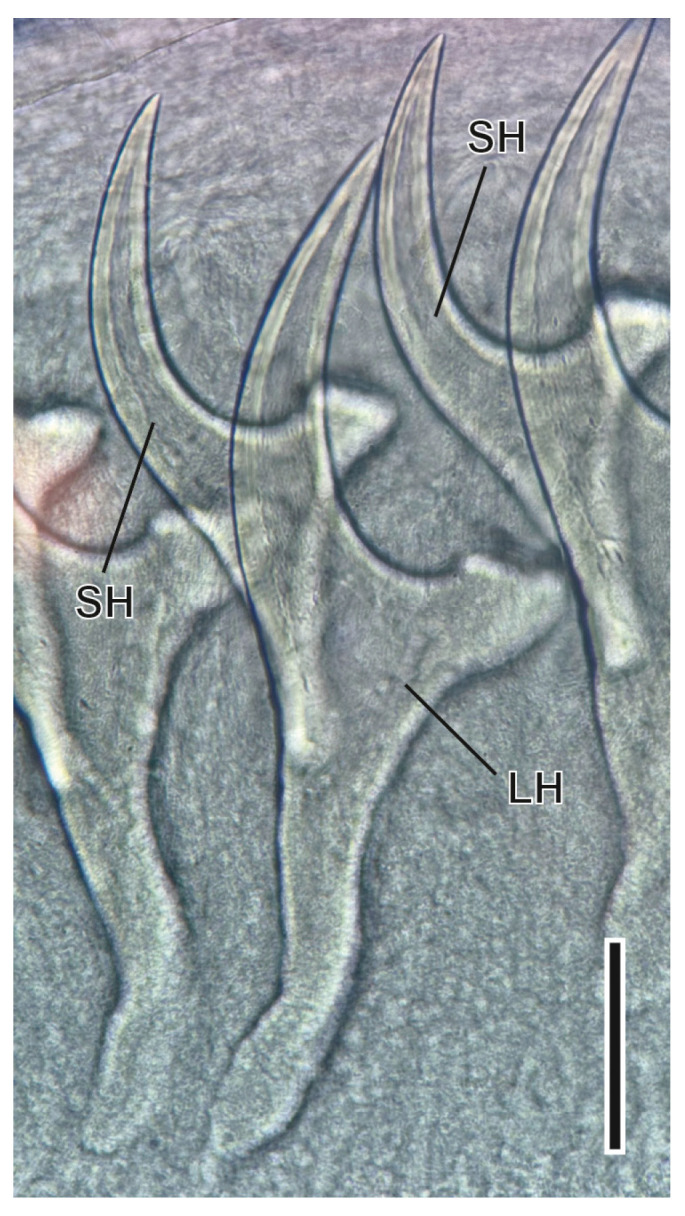
Detail of large (LH) and small hooks (SH) of *Hydatigera taeniaeformis* s.l. larvae. Scale bar = 100 µm.

#### 3.1.2. *Aonchotheca wioletti* (Rukhlyadeva, 1950)

This Capillaridae nematode was found parasitising the stomach. Males have a pair of small caudal lateral alae (12–22 μm/17–30 μm) and a narrow and filiform spicule (279–309 µm/9–43 μm). In females, the vulva is situated 21–43 µm from the basis of the oesophagus and 1.53–3.15 mm from the cephalic end. Eggs present a typical lemon shape (60 μm/26–30 μm) [48]. Although it has not been demonstrated, the life cycle of this nematode is supposed to be diheteroxenous, with earthworms acting as the intermediate host carrying the larval stage infective for the montane water vole.

#### 3.1.3. *Eucoleus bacillatus* (Eberth, 1863) (Figure 4)

This other Capillaridae nematode was also found parasitising the stomach. Males and females present two bacillar bands along their body [49]. The caudal end of the males is ventrally curved, presenting a bursa with two lateral lobes, and the cloaca is terminal. They have a very fine spicule (774 µm/3 µm) surrounded by a sheath 395–456 µm long and covered by strong spines that are 2–3 µm long. In the females, the distance from the vulva to the oesophagus is 198 µm. Eggs have an elongated shape, with an ornamented cover (61.2–68.4 µm/28.8–32.4 µm). Its life cycle is monoxenous of the pseudogeohelminth type with the infective form being an egg which embryonates in the environment.

**Figure 4:**
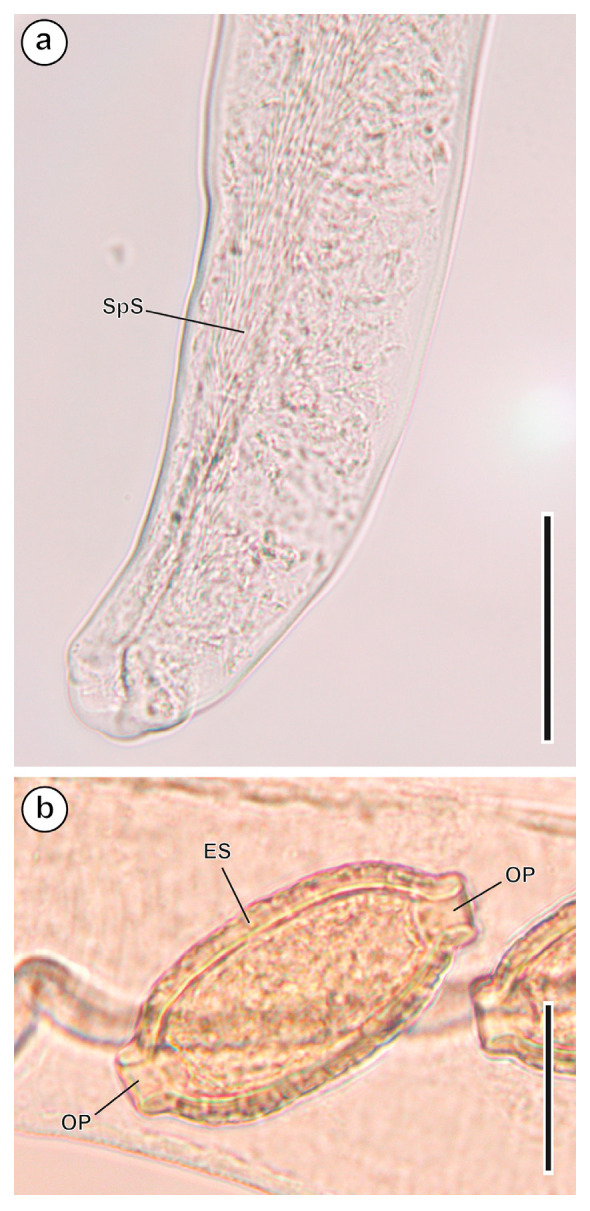
*Eucoleus bacillatus*. (**a**) Posterior extremity of a male. (**b**) Egg. ES, eggshell; OP, opercular plugs; SpS, spicular sheath. Scale bars: (**a**) = 200 µm; (**b**) = 25 µm.

#### 3.1.4. *Trichuris arvicolae* Feliu et al., 2000 (Figure 5)

This Trichuridae nematode was found parasitising the caecum. The morphology of the oesophagus, as well as the morphology and morphometry of female and male specimens, allowed the description of this nematode as a new species [50]. The ratio between the oesophageal and postoesophageal region in the males is 1.62–2.94, while it is 1.36–2.71 in females. Males present a fine spicule with a rounded distal end surrounded by a spiny sheath. Females have a muscular vagina that is 200–700 µm long. Eggs are elongated with two polar plugs (67–79 µm/31–43 µm). Its life cycle is monoxenous of the pseudogeohelminth type.

**Figure 5:**
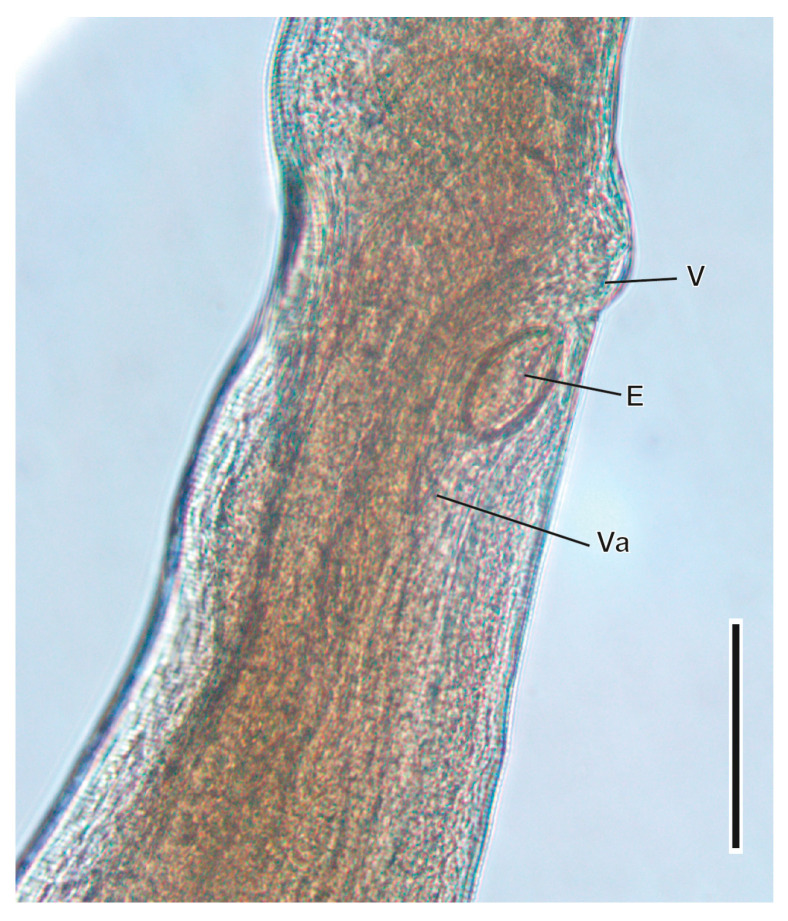
Vulvar region of a female of *Trichuris arvicolae*. E, egg; V, vulva; Va, vagina. Scale bar = 100 µm.

#### 3.1.5. *Carolinensis minutus* (Dujardin, 1845) (Figure 6)

This heligmosomid nematode was found parasitising the small intestine. It is characterised by its very small size, the presence of longitudinal ridges covering its body, the vesicle covering its head, and the caudal bursa of male specimens [51].

**Figure 6:**
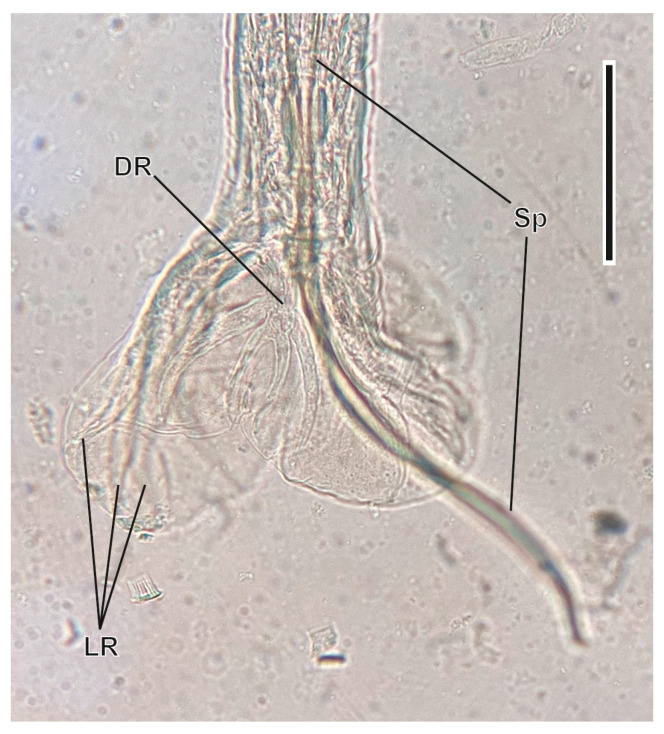
Caudal bursa of a male of *Carolinensis minutus*. DR, dorsal ray; LR, lateral rays; Sp, spicule. Scale bar = 1 µm.

The posterior part in males ends in a bilobulated copulatory bursa, being symmetric and without prebursal papillae. The spicules are elongated, being 280–350 µm long, while the gubernaculum is leaf-shaped, being 27–34 µm long. The vulva of females is situated 90–120 µm from the posterior end; the tail presents two small papillae at the tip. Eggs measure 68–85 µm/37–53 µm. Its life cycle is monoxenous of the geohelminth type with the infective form being a free larval stage in the environment, which either is ingested by the montane water vole or penetrates its skin.

#### 3.1.6. *Syphacia nigeriana* Baylis, 1928 (Figure 7)

This Oxyuridae nematode was found parasitising the caecum. The oxyuriform oesophagus is one of the most relevant characteristics of the genus. The morphology and morphometry of the sexual organs of males and females allow the identification of this nematode at the specific level [51]. Males present three ventral cuticular swellings situated 500, 750 and 1000 µm from the cephalic end; the spicules and the gubernaculum measure 68–75 µm and 27–31 µm, respectively. The vulva of females is situated 630–840 µm from the anterior end; the tail measures 530–700 µm. Eggs have a flattened side and measure 95–123 × 30–37 µm. Its life cycle is monoxenous of the ageohelminth type with the infective form being an embryonated egg, which is infective once released.

**Figure 7:**
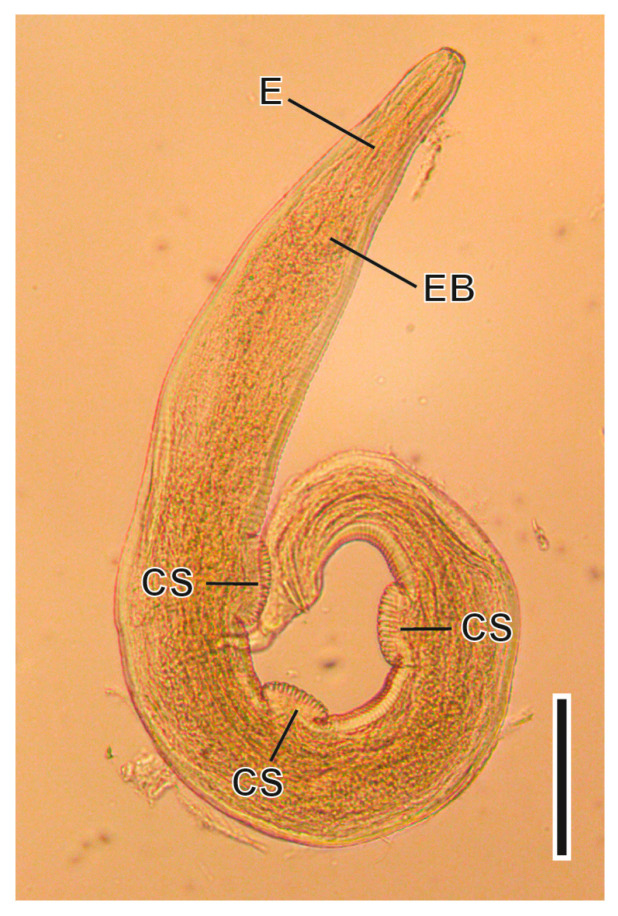
Male of *Syphacia nigeriana*. CS, ventral cuticular swellings; E, oesophagus; EB, oesophageal bulb. Scale bar = 150 µm.

### 3.2. Helminth Community Analysis

The four most prevalent species, *C. minutus*, *H. taeniaeformis* s.l. larvae, *S. nigeriana* and *T. arvicolae*, were analysed considering the sex, age, and season and year of capture of the host (Table 5). Although no significant differences were observed with respect to the host sex and the year of capture regarding the prevalence and abundance of these four helminth species, some results regarding the influence of host age and season of capture should be highlighted, such as the absence of *H. taeniaeformis* s.l. and *T. arvicolae*, and the low presence of *S. nigeriana* in the juvenile subpopulation, which contrasts with the higher prevalence of this subpopulation in the case of *C. minutus*, and the greater values of parasitism of *C. minutus* in the spring season and of *S. nigeriana* in both autumn and summer.

#### 3.2.1. Helminth Community Components

Infracommunity species richness of helminths ranged from one to three species: 379 hosts (46.50%) were parasitised by a single helminth species, while only 78 (9.57%) and 7 (0.85%) hosts presented infracommunities composed of two or three helminth species, respectively. The abundance index values make it possible to establish the following community structure: *C. minutus* (AI = 3.12) and *S. nigeriana* (AI = 3.11) as dominant species; *T. arvicolae* (AI = 0.12) as a co-dominant species; *E. bacillatus* (AI = 0.01) as a successful immigrant species; and *A. wioletti* (AI = 0.00) as a non-successful immigrant species. The larval stage of *H. taeniaeformis* is not included in this classification as *A. scherman* acts as an intermediate host for this cestode. The frequency distribution of the most prevalent species, expressed by the Lefkovitch index values, showed that *S. nigeriana* (L = 0.993) and *C. minutus* (L = 0.974) presented a high aggregated distribution, but moderate aggregation was present in the case of *T. arvicolae* (L = 526) and *H. taeniaeformis* s.l. larvae (L = 0.429).

#### 3.2.2. Helminth Community Diversity

To study the diversity/uniformity of the helminth community, the Shannon, Simpson, Berger–Parker and Shannon evenness indices were calculated, considering the total host population and the subpopulations determined by host age and sex as well as season and year of capture (Table 6). Males presented slightly higher values than females. Moreover, diversity increased with host age, with clear differences between the age subpopulations. Individuals captured in spring and winter as well as those captured during the 2012–2013 period showed the highest diversity values.

#### 3.2.3. Helminth Infracommunity Diversity

Diversity characteristics of the helminth infracommunities, according to host sex and age, and season and year of capture, confirm the tendency reported in the analysis of the helminth diversity community (Table 7). Infracommunities of *A. scherman* subpopulations of adults and of voles captured in spring and during the 2012–2013 period have a higher diversity than the other vole subpopulations. Once again, host sex does not determine any difference at the infracommunity diversity level.

#### 3.2.4. Effects of Host Age and Sex, and Season and Year of Capture on Helminth Infection Parameters

The analysis of extrinsic (climatology, meteorological station, and season and year of capture) and intrinsic (host age and sex) factors in the parasitation of the component helminth species revealed that most of these factors significantly influenced at least one species (Table 8 and Table 9). Specifically, host age had a significant effect on *H. taeniaeformis* larvae, *C. minutus*, and *T. arvicolae*, whereas climatology significantly affected *A. Scherman* and *S. nigeriana*. The influence of host age on the abundance of *C. minutus* is reaffirmed by the only significant result obtained from the statistical analysis of mean intensity (H = 23.445; df = 2; *p* < 0.0001). In addition, combinations of host age with meteorological station or season of capture further contributed to variation in parasitation values for *T. arvicolae* and *C. minutus*. Notably, host sex did not have a significant effect in any of the models. These results indicate that both intrinsic and extrinsic factors play important roles in shaping helminth infection patterns across the host population.

The analysis of correlations between the total prevalence and abundance of the helminth community, as well as of each of the helminth component species, carried out by the Spearman correlation coefficient, showed that the only significant results obtained were those between the total prevalence of the helminth community and mean temperature (r_s_ = −0.821; *p* = 0.023), as well as the prevalence of *S. nigeriana* and mean temperature (r_s_ = −0.717; *p* = 0.030), besides mean and accumulated rainfall (r_s_ = −0.717; *p* = 0.030). It is worth mentioning that these three correlations are negative and that there is no correlation between climate factors and diversity parameters.

### 3.3. Association Between Body Condition and Parasite Burden

Pearson correlation analyses revealed generally weak associations between body condition (BCI) and parasitological variables. Parasite species richness showed a slight positive correlation with BCI (r = 0.17; *p* = 4.55 × 10^−4^), suggesting a marginal tendency for individuals in better body condition to host a higher number of parasite species. In contrast, most measures of parasite burden exhibited weak negative correlations with BCI, including *Carolinensis minutus* (r = −0.34; *p* = 8.75 × 10^−8^), total parasitism (r = −0.14; *p* = 6.72 × 10^−5^), and total nematode abundance (r = −0.22; *p* = 7.68 × 10^−10^). These values indicate a slight tendency for higher parasite loads to be associated with poorer body condition, although the overall effect sizes were small and relationships were highly variable.

**Figure 8:**
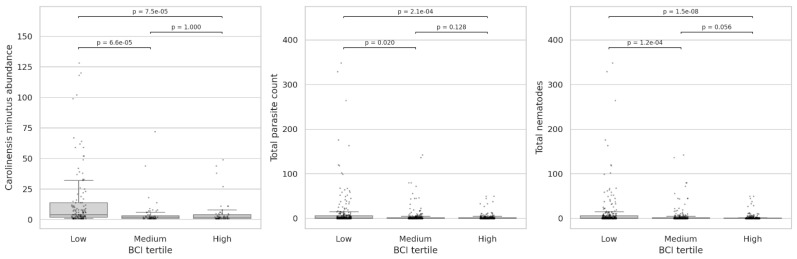
Box-plots showing BCI tertiles for fossorial water vole (*Arvicola scherman*) in relation to *Carolinensis minutus* abundance, total parasitism (helminth count), and total nematodes. The dark line in the middle of the boxes shows the median, and the bottom and the top of the box indicate the 25th and 75th percentile, respectively. The T-bars that extend from the boxes mark the minimum and maximum values. Pairwise Mann–Whitney comparisons with Holm–Bonferroni-corrected *p*-values are shown in each case.

Non-parametric analyses based on BCI tertiles revealed clearer patterns. Parasite species richness differed significantly among low-, medium-, and high-BCI groups (Kruskal–Wallis, *p* = 0.0013), with individuals in the higher-BCI category exhibiting slightly greater richness than those in lower condition. Stronger associations were observed for parasite abundance metrics. *Carolinensis minutus* abundance varied markedly across BCI tertiles (Kruskal–Wallis, *p* = 1.3 × 10^−6^), with substantially higher counts in individuals with low BCI (Figure 8). Similarly, total helminth burden showed a significant gradient across BCI groups (Kruskal–Wallis, *p* = 2.5 × 10^−4^), and total nematode abundance exhibited an extremely strong association with body condition (Kruskal–Wallis, *p* = 1.0 × 10^−8^), with the highest nematode loads consistently observed in low-BCI individuals (Figure 8). In contrast, no clear relationship with BCI was detected for several individual parasite species. *Hydatigera taeniaeformis* s.l., *T. arvicolae*, and *S. nigeriana* did not show significant associations with body condition in the non-parametric analyses (*p*_value > 0.05 in all cases), while *E. bacillatus* and A. wioletti could not be meaningfully evaluated due to their very low prevalence.

Overall, these results indicate that higher parasite burden, particularly total helminth and nematode abundance, is strongly associated with poorer body condition. This pattern is driven primarily by *C. minutus* and by aggregated measures of parasitism, whereas several individual parasite species do not exhibit independent relationships with host body condition.

## 4. Discussion

### 4.1. Helminth Community Analysis

The analysis of the *A. scherman cantabriae* helminth community from Asturias showed that (1) dominant species showed a low host specificity; (2) infracommunities presented low values of species richness, which can be explained by the underground life and the herbivorous diet of this vole [22,52]. The latter result also agrees with the low prevalences and the values of the diversity indices.

This is not the first time that the helminthfauna of Iberian *A. scherman* has been assessed. Previous studies in the Iberian Peninsula [21,22] concerning *A. scherman* (then referred to as *A. terrestris*) were carried out, analysing specimens from various Spanish locations. The capture sites of the individuals recorded in these studies have enabled us to assign them to one of the two subspecies of *A. scherman* recognised in the Iberian Peninsula, *A. scherman cantabriae* and *A. scherman monticola*. Thus, the helminthfauna reported for *A. scherman cantabriae* consisted of a total of six helminth species: the cestode *Paranoplocephala omphalodes*, and five nematodes, *A. wioletti*, *T. arvicolae* (although reported as *Trichuris* sp.), *Heligmosomoides laevis*, *H. glareolus* and *S. nigeriana*. Moreover, the helminthfauna found in *A. scherman monticola* consisted of a total of ten helminth species: five cestodes, the larval stage of *H. taeniaeformis* s.l. and the adults of *Anoplocephaloides dentata*, *P. omphalodes*, *Eurotaenia gracilis* and *Arostrilepis horrida* (=*Hymenolepis horrida*), and five nematodes, *A. wioletti*, *E. bacillatus*, *T. arvicolae* (reported as *Trichuris* sp.), *H. laevis* and *S. nigeriana*. Although the structure of the helminth community reported in the present study for *A. scherman* is similar to that found in other Iberian Arvicolinae [22,26], there are some noteworthy differences compared with the previous studies in the Iberian Peninsula mentioned above [21,22]. The prevalence of infection of *A. scherman cantabriae* in Asturias (57%) does not differ greatly from that of *A. scherman monticola* in the Spanish Pyrenees (58%). In this sense, it is worth highlighting the high number of individuals analysed of both subspecies of water voles, *A. scherman cantabriae* (n = 815) and *A. scherman monticola* (n = 469) [21], which allows us to obtain more reliable prevalence results, which, in this case, are practically the same.

Concerning tapeworm helminths, two facts should be highlighted. First, cestodes for which *A. scherman cantabriae* from Asturias acts as the definitive host are not reported, while four species were reported in *A. scherman monticola*, and one species in *A. scherman cantabriae*. However, all adult tapeworms had low prevalences, probably since their intermediate hosts are arthropods. In the case of Asturias, the pesticides used in the farmland of the study area could explain the absence of adult tapeworms. Second, the larval stage of *H. taeniaeformis* s.l. parasitising *A. scherman cantabriae* is reported for the first time. Comparative analysis indicates that this cestode shows a higher prevalence in this subspecies than in *A. scherman monticola*. In the study area, cats, the definitive host of this taeniid species, abound, which facilitates infestation of *A. scherman cantabriae* with the tapeworm eggs. This also happens in the Iberian pine vole, *Microtus lusitanicus*, another arvicoline species that is present in this area [26].

This study also constitutes the first record of *E. bacillatus* parasitising *A. scherman cantabriae* (0.4%). This capillarid was reported before in *A. scherman monticola* from Lleida but with a very high prevalence of 50%. *Eucoleus bacillatus* is a relatively unimportant species in the helminthfauna of *A. scherman cantabriae*; the high number of individuals analysed allowed its detection, but in three individuals only. In the *A. scherman monticola* population of the Pyrenees, the prevalence is much higher, probably due to both the better climatic conditions for the completion of the biological cycle and the greater proximity of populations of the wood mouse, *Apodemus sylvaticus* [53], a typical host of this capillarid.

The prevalence of *T. arvicolae* is higher than in *A. scherman monticola* but lower than in other populations of *A. scherman cantabriae* [21,22]. The higher prevalence than in the Pyrenees could be due to the presence of other host species such as *Microtus lusitanicus* in the study area, which also carried this nematode [26]; the lower prevalence than in other populations of *A. scherman cantabriae* is likely to be due to the high number of individuals analysed in the current study, and because it is not a dominant species.

Nematodes of the genus *Heligmosomoides* have not been detected in the vole population from Asturias. However, these nematodes are rare in *A. scherman monticola* but have a prevalence of 7–8.5% in other populations of *A. scherman cantabriae*. This is one of the rarest and most unexpected absences, especially considering the percentage of parasitism in other populations of *A. scherman cantabriae* [21,22].

This study also represents the first record of *C. minutus* in *A. scherman*, which is, moreover, the most prevalent species. This nematode also occurs in *Microtus lusitanicus*, a host that is also very abundant in the study area and in which *C. minutus* is also the most prevalent species [26]. Consequently, it can be stated that *C. minutus* is shared by some Arvicolinae hosts in the Iberian Peninsula.

Prevalence of *S. nigeriana* (12%) is higher than that in *A. scherman cantabriae* (3.4%) from other locations as well as in *A. scherman monticola* (1.0%). The montane water vole is considered a pest in the study area, and at the time of capture, its populations were highly aggregated. Thus, the mode of transmission of the pinworm may explain this high prevalence.

In general, the absence of helminth species that use invertebrates as their intermediate host, except for the less prevalent nematode *A. wioletti*, which have *A. scherman* as their definitive host, could be explained by habitat fragmentation [29,54], but also by the herbivorous diet of these voles.

#### 4.1.1. Helminth Community Components

If the hypothesis postulated by several authors [55,56,57] that “the aggregation of parasites is related to host-parasite stability, i.e., high values of aggregation mean more stability”, is accepted, it would indicate that *H. taeniaeformis* s.l. larvae and *T. arvicolae* have a destabilising role in the *A. scherman* populations analysed. In contrast, the two dominant species, *C. minutus* and *S. nigeriana*, presented high aggregation values, probably due to several factors, such as the heterogeneity of host populations and/or parasitism pressure [58]. The level of aggregation does not appear to be related to the parasite’s transmission mechanism or route, but it does reflect the optimal distribution of species and coevolutionary processes between parasites and their hosts [59]. Consequently, because of the potential to regulate *A. scherman* populations, *H. taeniaeformis* s.l. larvae and *T. arvicolae* could be proposed to be used in the control of montane water vole populations when required. It should also be noted that the tapeworm larva establishes itself on a vital organ such as the liver, and that *T. arvicolae* is the largest helminth of the helminthfauna and can produce a significant pathology in its host.

#### 4.1.2. Effects of Host Age and Sex, and Season and Year of Capture on Helminth Infection Parameters

Host age and other factors such as season and year of capture influence the diversity of both the helminth community and the infracommunities, as observed in the variability of the diversity index values, which are higher in the subpopulations of adult individuals and those captured during spring and summer and in the 2012–2013 period. However, it should be noted that all diversity index values, including the one calculated for the infracommunities (Brillouin index), are very low, reflecting the low diversity as a consequence of the dominance of *C. minutus* over the rest of the species in terms of both prevalence and abundance, the latter parameter only being similar in the case of *S. nigeriana*.

According to the results of binary logistic regression and the Kruskal–Wallis test, the species influenced by host age are *H. taeniaeformis* s.l. larvae, *T. arvicolae*, *C. minutus*, and *S. nigeriana*.

In the case of *H. taeniaeformis* s.l. larvae, the increase in prevalence and abundance values with host age was expected. These results agree with previous studies in *A. terrestris*, currently *A. amphibius* or *A. scherman* [60,61], and other small mammal species [26,62,63,64,65,66,67,68,69,70,71,72,73,74,75,76,77]. Juvenile individuals are protected from egg infection by IgA ingested in colostrum and transplacental IgA and IgG [78]. Although adult hosts are also protected, they tend to be more infected as they cover larger territories and the time to be in contact with taeniid eggs, the infective form, in the environment is longer [79].

The prevalence and abundance of *T. arvicolae* also increased with age. These results differ from previous studies in which adults did not present any parasitation [80]. However, several studies in other hosts [26] and also in other *Trichuris* species show the same tendency observed herein where prevalence increases with age [81].

In *C. minutus*, the results were as expected: prevalence and abundance decrease with age, with the juvenile subpopulation of montane water voles being the most parasitised, in terms of both prevalence and abundance. One explanation could be that adult individuals have a more developed immune system, being more protected from infective forms than younger ones [66].

In *S. nigeriana*, although the highest prevalence corresponds to the adult subpopulation, preadult voles were the subpopulation with the highest abundance. This fact could be due, as mentioned above, to a more developed immune system in adult individuals [66]. However, previous studies carried out in other Arvicolinae species, which also host this oxyurid parasite, reported different tendencies between age groups [73,82,83].

The results indicate that host sex showed no effect on diversity, species richness, prevalences and abundances of the helminth community of *A. scherman*. This lack of a sex bias has also been reported in various small mammal species [26,67,68,69,78,84,85,86]. Some studies suggest that the influence of sex on the parasite load may depend largely on the parasite taxa and/or host–parasite associations [87,88,89,90]. Another study [86] hypothesises that a sex bias is related to a seasonally dependent sexual dichotomy in reproductive behaviour.

Results on the effect of season on diversity and species richness of infracommunities showed that the highest values correspond to spring, followed by summer, with the lowest values observed in autumn. As these two seasons are those with the highest outside activity of montane water voles, they are more likely to come into contact with parasite infective forms, a fact that could explain these values. Additionally, the low infection values in autumn might be more related to environmental conditions that influence the survival of dispersal stages. Summer could affect egg and larva survival negatively and, consequently, not many hosts would be infected in autumn. However, only *S. nigeriana* abundances were influenced by season. As this species is an ageohelminth nematode, transmission requires the host to be in contact with the embryonated released egg in the environment, while other routes of infection, such as autoinfection, grooming by other individuals or sharing nests, are also possible [83]. Other studies with different host species and different species of *Syphacia* showed a seasonal effect [26,72,83,91]. Since the montane water vole can reproduce year round under favourable conditions [32,92], the high values of abundance of *S. nigeriana* can be related to high levels of aggregation [91,93,94,95].

The year of capture influences both the diversity indices and species richness. These variables showed the highest values in 2012–2013. The abundance of *S. nigeriana* was the only parameter influenced by this factor, which could be explained by climatic differences and, once again, by the variability of the host density across the study period.

The lack of correlations between climate variables and the diversity indices of the infracommunities (species richness and Brillouin index), which remain unexplained, should be highlighted, especially considering that the four most prevalent species have as their infective form an egg or a larva present in the environment and that they might be influenced by the climatic variables of the study area. However, the overall prevalence of the helminth community is negatively influenced by the mean temperature, and the prevalence of *S. nigeriana* is negatively affected by both mean and cumulative rainfall. Temperature can influence the viability of infective forms, thus affecting the helminth prevalence, but the influence of rainfall on the prevalence of *S. nigeriana*, an ageohelminth, cannot be explained, as noted above, because no correlations were found for any other species, such as geohelminths or pseudogeohelminths, whose infective forms do depend on rainfall for their survival.

### 4.2. Association Between Body Condition and Parasite Burden

The relationship between helminth infection and host body condition in *A. scherman* revealed in this study appears to be complex and strongly context-dependent. Although Spearman correlation analyses indicated generally weak associations between body condition and parasitological variables, non-parametric analyses based on BCI categories revealed clearer and biologically meaningful patterns. In particular, individuals with a poorer body condition consistently harboured higher total helminth and nematode burdens, a pattern largely driven by *C. minutus*. This finding supports the hypothesis that aggregated parasite loads, rather than the presence of individual species per se, are more relevant for detecting parasite-mediated effects on the physiological status of the host [23,24]. Similar patterns have been reported in other wild rodent systems, where high helminth burdens are associated with reduced energetic reserves and a compromised condition, even when individual parasite species show weak or inconsistent effects [27]. Interestingly, parasite species richness showed a slight positive association with BCI, suggesting that individuals in a better condition may experience a higher degree of parasitism due to increased exposure associated with their healthier status and more active behaviour [27]. In contrast, the weak or non-significant relationships observed for several parasite taxa, including *H. taeniaeformis* s.l. and *T. arvicolae*, may reflect differences in pathogenicity, infection intensity, or life-cycle characteristics, as well as a relatively high tolerance of hosts to low or moderate infection levels [23,24]. Overall, these results indicate that helminth infection, particularly nematode abundance, may contribute to a reduced body condition in *A. scherman*, thus potentially decreasing reproductive potential [10] and increasing vulnerability to predation, and may therefore be associated with processes influencing population density phases [16]. This supports the view that this vole species can be considered a suitable model for exploring parasite-mediated mechanisms in the dynamics of overabundant rodent populations, while also highlighting the need to interpret parasitological effects within a multifactorial ecological framework.

### 4.3. The Use of the Helminths of Arvicola scherman in Pest Control

The effects of parasites on the reproduction and survival of wild animal populations have become widely acknowledged [18,96,97,98,99]. In this sense, parasites could play an important role in regulating vertebrate populations [96,100,101]. Considering the results concerning the moderate aggregation of *H. taeniaeformis* s.l. larvae and *T. arvicolae*, these two helminth species could play a role in the destabilisation of montane water vole populations and consequently could be proposed to control populations of their hosts, as previously proposed in the study of *M. lusitanicus* in the same study area [26].

Several studies have reported that the larval stage of *H. taeniaeformis* may affect populations of small mammals. Lin et al. (1990) [102] demonstrated reduced fertility, mating success, and number of offspring carried to full term in rats infected with the larval stage of this tapeworm. These effects may intensify patterns observed in small mammal population cycles, either by extending periods of low density or by amplifying the severity of population declines. Dvorakova and Prokopic, 1984 [103], reported mass deaths of muskrats (*Ondatra zibethicus*) in the Czech Republic due to infection of the larval stage of *H. taeniaeformis*. Other studies [104] related the prevalence of the tapeworm to the population density of *A. terrestris*, observing higher levels of prevalence in low-density populations, but without concluding that the helminth can regulate the vole population. Future studies on the difference in body condition and fecundity in infected versus non-infected voles may reveal a potential influence of this tapeworm’s larval stage on the regulation of small mammal populations.

Regarding *T. arvicolae*, studies carried out by Deter et al. [105,106] showed that this nematode, which affects the fecundity of its host, could control population growth of infected rodents. Information obtained through models in populations of *A. terrestris* [106] showed that this intestinal nematode exerts a regulatory effect on the arvicoline population, with a reduction, according to the model, of 50.2% in the host population size.

Based on both the previous comments and our findings, *H. taeniaeformis* s.l. and *T. arvicolae* may exert a destabilising influence on *A. scherman* populations. Given their role in regulating host populations, both species could be considered as potential biological control agents for montane water vole populations in Asturias and other parts of their geographic range.

## 5. Conclusions

The helminthfauna of *A. scherman cantabriae* in NW Spain shows low diversity but is comparable to that of other Iberian populations. However, the loss of some helminth species is observed, likely due to pesticide use and habitat fragmentation. Overall, the helminthfauna is poorer than that of *A. scherman monticola*, reinforcing their taxonomic distinction. Host age is the most important factor influencing parasitism by the most prevalent species. In contrast, seasonal and climatic effects are more limited. These effects are mainly reflected in *S. nigeriana*, associated with host aggregation in areas of high population density. A high helminth burden, especially of aggregated species, is linked to poorer body condition. *Hydatigera taeniaeformis* s.l. and *T. arvicolae* may help regulate populations and act as biological control agents.

## Figures and Tables

**Table 1:** Distribution of *Arvicola scherman* population analysed by host sex and age.

Host Sex	Season	Host Age			
		Juveniles	Preadults	Adults	Total
Males	Winter	8	20	58	86
	Spring	16	23	77	116
	Summer	8	25	64	97
	Autumn	13	26	63	102
	Total	45	94	262	401
Females	Winter	5	20	58	83
	Spring	14	24	73	111
	Summer	17	16	67	100
	Autumn	12	31	77	120
	Total	48	91	275	414

**Table 2:** Distribution of *Arvicola scherman* population analysed by year and season of capture.

Year	Season				
	Winter	Spring	Summer	Autumn	Total
2011	67	124	109	127	427
2012	102	103	88	79	372
2013	16	0	0	0	16
Total	185	227	197	206	815

**Table 3:** Number of *Arvicola scherman* individuals analysed (n) by season and year of capture and seasonal values of climate variables corresponding to the year prior to host capture.

	n	Mean Temperature (°C)	Mean Rainfall (mm)	Cumulative Rainfall (mm)
Winter 2011	25	10.1	3.0	269.0
Spring 2011	92	15.0	4.0	338.4
Summer 2011	110	19.9	0.7	65.9
Autumn 2011	116	12.6	5.5	503.4
Winter 2012	101	11.1	2.9	246.0
Spring 2012	95	16.2	1.0	88.5
Summer 2012	86	19.7	1.3	116.2
Autumn 2012	72	14.6	2.2	199.0
Winter 2013	13	10.2	1.4	125.1

**Table 4:** Selected characteristics and parameter values of the helminth community of 815 individuals of *A. scherman* analysed.

Helminth Species	Site	Life Cycle	n	Prevalence (95% CI)	Mean Abundance (SE)	Mean Intensity (Range)
CESTODA						
*H. taeniaeformis* s.l. larvae	L	DH	155	19 (16–22)	0.25 (0.02)	1 (1–13)
NEMATODA						
*A. wioletti*	S	DH	1	0.1 (0.0–0.5)	<0.00 (<0.00)	2 (2)
*E. bacillatus*	S	M-P	3	0.4 (0.0–0.5)	0.01 (<0.00)	1 (1–3)
*T. arvicolae*	C	M-P	57	7 (5–9)	0.12 (0.02)	1 (1–8)
*C. minutus*	I	M-G	246	30 (27–37)	3.12 (0.42)	3 (1–128)
*S. nigeriana*	C	M-A	94	12 (10–14)	3.11 (0.79)	5 (1–348)

n, number of *A. scherman* parasitised; CI, confidence interval; SE, standard error; DH, diheteroxenous; M, monoxenous; A, ageohelminth; G, geohelminth; P, pseudogeohelminth; I, intestine; L, liver; C, caecum; S, stomach.

**Table 5:** Selected characteristics of the four most prevalent species by host age and sex, and season and year of capture.

			Species			
			*H. taeniaeformis* s.l. Larvae	*T.* *arvicolae*	*C.* *minutus*	*S.* *nigeriana*
Sex	Males	Prevalence (CI 95%)	21 (17–25)	6 (4–9)	31 (27–36)	11 (8–14)
		Mean abundance (SE)	0.27 (0.04)	0.11 (0.03)	2.97 (0.55)	2.39 (0.63)
		Mean intensity (range)	1 (1–13)	1 (1–8)	3 (1–128)	6 (1–264)
	Females	Prevalence (CI 95%)	17 (14–21)	8 (6–11)	29 (25–34)	12 (9–15)
		Mean abundance (SE)	0.22 (0.03)	0.12 (0.03)	3.27 (0.55)	3.82 (0.83)
		Mean intensity (range)	1 (1–4)	1 (1–6)	3 (1–120)	5 (1–348)
Age	Juveniles	Prevalence (CI 95%)	-	-	54 (44–84)	4 (1–10)
		Mean abundance (SE)	-	-	12.18 (2.86)	1.49 (0.87)
		Mean intensity (range)	-	-	6 (1–128)	36 (3–64)
	Preadults	Prevalence (CI 95%)	8 (5–13)	6 (3–10)	44 (37–51)	10 (6–15)
		Mean abundance (SE)	0.16 (0.08)	0.10 (0.03)	4.24 (0.81)	6.74 (3.11)
		Mean intensity (range)	1 (1–13)	1 (1–3)	3 (1–67)	2 (1–348)
	Adults	Prevalence (CI 95%)	26 (13–25)	8 (6–11)	21 (18–25)	13 (10–16)
		Mean abundance (SE)	0.32 (0.03)	0.14 (0.03)	1.16 (0.23)	2.15 (0.51)
		Mean intensity (range)	1 (1–4)	1 (1–8)	2 (1–72)	6 (1–142)
Season	Winter	Prevalence (CI 95%)	18 (13–25)	7 (4–12)	25 (19–32)	9 (5–14)
		Mean abundance (SE)	0.20 (0.04)	0.13 (0.04)	1.89 (0.56)	3.19 (2.02)
		Mean intensity (range)	1 (1–4)	1 (1–6)	2 (1–64)	7 (1–348)
	Spring	Prevalence (CI 95%)	21 (1–4)	10 (6–15)	48 (41–55)	8 (5–12)
		Mean abundance (SE)	0.26 (0.03)	0.09 (0.05)	5.91 (1.05)	3.39 (1.88)
		Mean intensity (range)	1 (1–3)	1 (1–8)	3 (1–118)	6 (1–329)
	Summer	Prevalence (CI 95%)	21 (16–27)	5 (2–9)	29 (23–36)	13 (16–27)
		Mean abundance (SE)	0.26 (0.08)	0.09 (0.03)	3.41 (1.08)	2.91 (1.01)
		Mean intensity (range)	1 (1–13)	2 (1–3)	3 (1–128)	7 (1–142)
	Autumn	Prevalence (CI 95%)	16 (12–21)	6 (3–10)	16 (12–21)	16 (12–21)
		Mean abundance (SE)	0.02 (0.04)	0.07 (0.02)	0.87 (0.26)	2.94 (1.12)
		Mean intensity (range)	1 (1–3)	1 (1–2)	3 (1–38)	3 (1–176)
Year	2011	Prevalence (CI 95%)	18 (15–22)	6 (4–9)	28 (24–32)	7 (5–10)
		Mean abundance (SE)	0.22 (0.03)	0.11 (0.03)	3.21 (0.68)	1.29 (0.35)
		Mean intensity (range)	1 (1–3)	1 (1–8)	3 (1–128)	10 (1–80)
	2012–2013	Prevalence (CI 95%)	20 (16–24)	9 (6–12)	32 (28–37)	17 (14–21)
		Mean abundance (SE)	0.27 (0.04)	0.13 (0.02)	3.02 (0.45)	5.12 (1.61)
		Mean intensity (range)	1 (1–13)	1 (1–4)	3 (1–72)	3 (1–348)

CI, confidence interval; SE, standard error.

**Table 6:** Diversity characteristics of the helminth community of *Arvicola scherman* by host age and sex, and season and year of capture.

		Shannon Index	Simpson Index	Berger–Parker Index	Shannon Evenness Index
Sex	Males	0.93	0.56	0.48	0.58
	Females	0.89	0.54	0.49	0.49
Age	Juveniles	0.35	0.19	0.11	0.31
	Preadults	0.78	0.50	0.40	0.49
	Adults	1.03	0.57	0.43	0.58
Season	Winter	0.90	0.53	0.41	0.56
	Spring	0.85	0.51	0.39	0.48
	Summer	0.91	0.55	0.49	0.66
	Autumn	0.78	0.43	0.28	0.57
Year	2011	0.85	0.48	0.34	0.64
	2012–2013	0.86	0.52	0.40	0.48
Total		0.91	0.55	0.55	0.51

**Table 7:** Diversity characteristics of helminth infracommunities of *Arvicola scherman* by host sex and age, and season and year of capture.

		Mean Species Richness X/S.E.	Brillouin Index (B.I.) X/S.E./Max.	B.I. of Infected *A. scherman* Only X/S.E.	% of *A. scherman* Infected
Sex	Males	0.69/0.04	0.04/0.01/0.72	0.07/0.01	56.86
	Females	0.67/0.03	0.03/0.01/0.68	0.06/0.01	57.00
Age	Juveniles	0.58/0.03	0.01/0.01/0.50	0.02/0.01	54.83
	Preadults	0.69/0.05	0.03/0.01/0.58	0.04/0.01	61.08
	Adults	0.70/0.03	0.04/0.01/0.72	0.08/0.01	55.87
Season	Winter	0.59/0.05	0.02/0.01/0.43	0.05/0.01	51.89
	Spring	0.89/0.05	0.06/0.01/0.72	0.08/0.01	70.04
	Summer	0.69/0.05	0.04/0.01/0.58	0.07/0.01	56.85
	Autumn	0.53/0.04	0.02/0.01/0.45	0.04/0.01	47.09
Year	2011	0.59/0.03	0.03/<0.00/0.67	0.05/0.01	51.76
	2012–2013	0.78/0.04	0.05/0.01/0.72	0.08/0.01	62.63
Total		0.68/0.02	0.04/<0.00/0.72	0,06/0.01	56.93

S.E, standard error.

**Table 8:** Binary logistic regression models for the prevalence of the helminth component species of *Arvicola scherman* by year and season of capture, host sex and age, climatology, and meteorological station expressed by ꭓ^2^ values with associated probabilities (*p*) for the model created, including independent variables. Only statistically significant models are reported.

Helminth Species/Independent Variables Included in the Model	df	ꭓ^2^	*p*
Global *A. scherman* parasitation			
Climatology	8	77.778	<0.0001
*H. taeniaeformis* s.l. larvae			
Host age	2	72.969	<0.0001
*T. arvicolae*			
Meteorological station	1	37.498	<0.0001
Meteorological station and host age	3	51.024	<0.0001
*C. minutus*			
Host age	2	57.926	<0.0001
Host age and season of capture	5	125.393	<0.0001
*S. nigeriana*			
Climatology	8	33.883	<0.0001
Climatology and meteorological station	9	40.268	<0.0001

df, degrees of freedom.

**Table 9:** Values of Mann–Whitney (U) and Kruskal–Wallis (H) tests, with associated probabilities (*p*), applied in the analyses of the helminth component species abundances of *Arvicola scherman*. The Mann–Whitney test was applied to host sex and the Kruskal–Wallis test was applied to host age, and season and year of capture. Only significant results are reported.

Helminth Species/Independent Variables Included in the Model	df	U/H	*p*
*H. taeniaeformis* s.l. larvae			
Host age	2	52.731	<0.0001
*T. arvicolae*			
Host age	2	8.602	0.014
*C. minutus*			
Host age	2	74.672	<0.0001
*S. nigeriana*			
Host age	2	6.307	0.043
Year of capture	1	75,204	<0.0001
Season of capture	3	8.049	0.045

df, degrees of freedom.

## Data Availability

The database used to carry out the present study is not publicly available due to internal policy of our departments. However, the database could be made available, after a justification of its use, upon request from the corresponding author.
